# Integrated Spinal and Supraspinal Mechanisms of Spinal Cord Stimulation Analgesia: A Systematic Review of Bidirectional Neural Modulation

**DOI:** 10.7759/cureus.90434

**Published:** 2025-08-18

**Authors:** Muhammad Usman, Hideaki Yamamoto, Jaden Y Fang, Adam Romman, Aristides P Koutrouvelis, Satoshi Yamamoto

**Affiliations:** 1 Anesthesiology, University of Texas Medical Branch at Galveston, Galveston, USA; 2 Biological Sciences, University of California San Diego, San Diego, USA

**Keywords:** chronic neuropathic pain, chronic pain management, failed back surgery syndrome (fbss), spinal cord stimulation (scs), systematic review

## Abstract

Spinal cord stimulation (SCS) is an established therapy for refractory neuropathic pain, yet its spinal-level gating and supraspinal modulatory actions have rarely been unified into a single framework. Adhering to Preferred Reporting Items for Systematic Reviews and Meta-Analyses (PRISMA) guidelines, we conducted a comprehensive search and rigorous selection of animal studies that investigated both spinal and supraspinal circuitry involved in SCS-induced analgesia. Through this process, we identified and systematically reviewed seven rodent studies employing tonic, burst, or sub-perception SCS in mononeuropathy and spared-nerve-injury models, extracting electrophysiological, molecular, lesion, and imaging data. Across these models, SCS consistently restored dorsal-horn inhibitory tone, enhancing GABAergic and glycinergic signaling, suppressing wide-dynamic-range neuron hyperactivity, and attenuating mitogen-activated protein kinase (MAPK) and microglial activation. At the same time, epidural stimulation sent signals up the spinal cord that activated key brainstem pain-control centers: the periaqueductal gray, rostroventromedial medulla, and locus coeruleus. It also normalized abnormal cortical oscillations in somatosensory and limbic regions. Lesion and receptor-blockade experiments revealed that disruption of either ascending afferents or descending tracts halved analgesic efficacy, confirming a bidirectional feedback loop. Functional MRI further showed that burst versus tonic waveforms differentially recruit sensory versus affective networks. Together, these findings support a unified spinal-supraspinal model of SCS-mediated analgesia and highlight opportunities to refine electrode placement, waveform design, and stimulation parameters. While translational implications are tempered by variability in preclinical study designs, this integrated framework provides a roadmap for next-generation, mechanism-driven neuromodulation therapies tailored to individual pain needs.

## Introduction and background

Spinal cord stimulation (SCS) has become an established therapy for treating a variety of chronic pain syndromes, including persistent spinal pain syndrome type 2 (PSPS-T2), failed back surgery syndrome (FBSS), complex regional pain syndrome, and peripheral neuropathies [1]. Clinically introduced in the 1960s, SCS was originally conceptualized through the gate-control theory of Melzack and Wall, proposing that activation of large-diameter dorsal-column afferents could inhibit nociceptive transmission in the spinal dorsal horn [2]. For decades, this spinal-focused model guided device development and parameter selection, with an emphasis on blocking pain signals at the spinal cord level by activating local inhibitory neurons. More recent preclinical and clinical investigations, however, have revealed that SCS engages supraspinal networks as an integral part of its analgesic action. Functional neuroimaging studies in patients demonstrate that epidural stimulation alters activity within somatosensory and prefrontal regions, suggesting that SCS not only modulates sensory discrimination but also impacts affective dimensions of pain [3].

While our understanding of SCS mechanisms continues to evolve, many studies to date have focused on either spinal-level or supraspinal-level effects of SCS. Early work in the field detailed segmental gating in the dorsal horn [4], while other investigations focused on brain-mediated pathways [5]. This more compartmentalized approach may limit insight into how spinal and supraspinal mechanisms interact during stimulation.

Several recent reviews have examined the spinal-level actions of SCS [6] and its role in postoperative pain modulation [7], while others have examined supraspinal engagement [8] or detailed translational animal-model insights into central sensitization [9,10]. However, there remains more to investigate in integrating both segmental dorsal-horn mechanisms and brain-mediated descending pathways into a unified framework. To help address this, we conducted a systematic review of seven experimental animal studies that investigated both spinal and supraspinal mechanisms of SCS in the context of neuropathic pain. By synthesizing data from molecular assays, electrophysiological recordings, neuroimaging, and lesion studies, our aim is to clarify the bidirectional feedback loop through which SCS produces analgesia across spinal and brain circuits, and to identify key principles for translational optimization of this therapy.

## Review

Methods

To assess the underlying integrated spinal and supraspinal mechanisms of SCS, we conducted a comprehensive search of experimental animal studies through prominent electronic medical information repositories, including PubMed, MEDLINE, Google Scholar, ScienceDirect, BMJ databases, and Cochrane databases. This systematic review was registered with the International Platform of Registered Systematic Review and Meta-analysis Protocols (INPLASY) (registration number: 202540047). The search flow diagram strictly follows the Preferred Reporting Items for Systematic Reviews and Meta-Analyses (PRISMA) guidelines. 

Search Strategy

A comprehensive search strategy was designed to identify experimental animal studies investigating the combined spinal and supraspinal mechanisms of SCS in the context of neuropathic pain. Searches were conducted in PubMed, ScienceDirect, Embase, and Web of Science databases, encompassing studies published from database inception through July 2025. The search queries are shown in the Appendix. 

Data Extraction

To ensure the thoroughness of the search, two reviewers (HY and JF) evaluated the articles independently to ascertain their adherence to the inclusion criteria. Any discrepancies were resolved through a third, independent reviewer. 

Study Selection

The literature search was limited to English-language articles. References of all eligible articles were also screened to identify additional relevant studies. Inclusion criteria were: experimental animal studies using spinal cord stimulation in models of neuropathic pain, studies that explicitly investigated both spinal and supraspinal effects or readouts, and studies that reported mechanistic data. Examples of such data include neuronal readouts (e.g., dorsal horn wide-dynamic-range neuron firing or receptive field changes), neurochemical measures (e.g., microdialysis of glutamate/GABA, spinal 5-HT or norepinephrine levels), and functional or systems-level markers (e.g., c-Fos mapping, fMRI activation patterns, lesion or receptor-blockade experiments). Studies that evaluated only spinal or only supraspinal mechanisms in isolation, or that focused solely on peripheral nerve stimulation, were excluded from the analysis. The final list of included studies was determined after full-text evaluation and cross-verification with the inclusion criteria. This process yielded seven core preclinical studies that investigated both spinal and supraspinal contributions to SCS-induced analgesia using a range of mechanistic endpoints. 

*Risk of Bias and Methodological Quality Assessment* 

To ensure the credibility of findings, each included study was evaluated using the Systematic Review Centre for Laboratory Animal Experimentation Risk of Bias (SYRCLE RoB) tool, which is specifically designed for animal experiments. The tool accesses the papers on random allocation of animals to experimental groups and reported baseline characteristics to mitigate selection bias, and whether investigators were blinded to group assignment during procedures and outcome measurement to address performance and detection biases. Completeness of outcome data was reviewed to identify potential attrition bias, while protocols and publications were compared to detect selective reporting. We also reviewed other design features, such as anesthesia protocols and stimulus calibration, for sources of systematic error unique to preclinical work. We also screened for the animal research: reporting of in vivo experiments (ARRIVE) guidelines to verify that essential experimental details, including animal species and strain, sex, age, surgical and stimulation methods, were transparently reported. Studies with low or unclear risk with SYRCLE and full compliance with ARRIVE were reported in this study. 

Results

Study Identification and Inclusion

**Figure 1:**
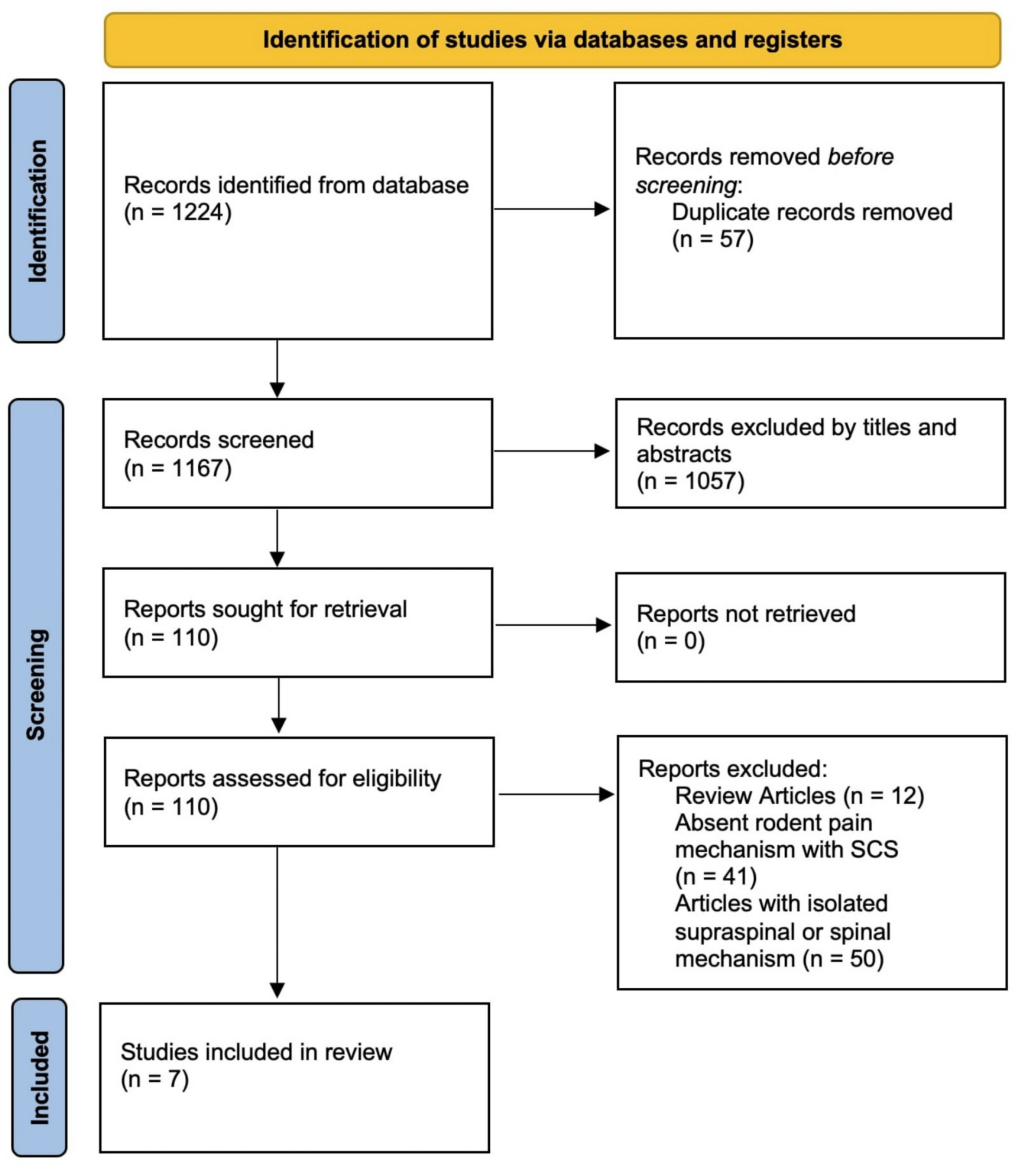
PRISMA flow diagram of the study selection process for pain mechanisms modulated by SCS in spinal and supraspinal regions of rodent models. PRISMA, Preferred Reporting Items for Systematic Reviews and Meta-Analyses; SCS, spinal cord stimulation

We initially screened 1224 texts that looked at the examination of pain mechanisms modulated by SCS in spinal cord cells of animal models. 57 duplicates were removed before continued screening. Of the remaining 1167 records, 1057 were excluded based on criteria from titles and abstracts. The residual 110 records were closely screened against the relevant exclusion criteria concerning systematic reviews, human subjects, and isolation of the proposed SCS mechanism to spinal or supraspinal only. Finally, seven full-text articles were selected for further in-depth analysis (Figure 1).

The studies included in this review are summarized in Table 1, which includes author(s) and publication year, species, treatment level, SCS parameters, pain model used, and conclusions. The studies are organized by publication year.

**Table 1:** Preclinical studies investigating the combined segmental and supraspinal mechanisms of spinal cord stimulation in animal models of neuropathic pain. SCS, spinal cord stimulation; T6, sixth thoracic vertebra; GABA, gamma-aminobutyric acid; c-Fos, cellular-Fos; PAG, periaqueductal gray; RVM, rostral ventromedial medulla; D2, dopamine D2 receptor; D3, dopamine D3 receptor; DRN, dorsal raphe nucleus; Hz, hertz; ACC, anterior cingulate cortex; 5-HT, 5-hydroxytryptamine; LC, locus coeruleus; fMRI, functional magnetic resonance imaging; S1, primary somatosensory cortex.

Author	Species	Treatment level	SCS parameters	Pain model	Conclusions
Ren et al., 1996 [11]	Female Sprague–Dawley rats	Ligation of the common sciatic nerve with further laminectomy at T6	10-minute stimulations: 50Hz pulses	Neuropathic Pain Model	SCS elevates the lowered threshold of the early A-fiber–mediated flexor reflex selectively in the nerve-ligated leg; the effect persists for 30–40 minutes after stimulation and is unchanged after T6 transection, indicating a spinal segmental mechanism without required supraspinal input.
Maeda et al., 2009 [12]	Adult Sprague–Dawley rats	Ligation and removal of tibial & common peroneal nerve stumps	30-minute stimulation: 4/60/100 Hz pulses ​	Neuropathic Pain Model	SCS induces frequency-dependent c-Fos expression in the spinal dorsal horn, PAG, and RVM; supports recruitment of ascending and descending pain modulatory circuits.
Barchini et al., 2012 [13]	Female Sprague–Dawley rats	Ligation of the tibial & peroneal nerve	5-minute pulses at 50Hz	Neuropathic Pain Model	SCS delivered rostrally or caudally suppresses mechanical and thermal hypersensitivity; dorsal column lesions reduce efficacy by about half, and blocking GABA, 5-HT, adrenergic, or D2/D3 signaling differentially weakens rostral versus caudal effects, indicating interacting segmental and supraspinal mechanisms.
Song et al., 2013 [14]	Male Wistar rats	Ligation and removal of tibial & common peroneal nerve stumps	30-minute stimulation: 50Hz pulses	Neuropathic Pain Model	SCS activates RVM serotonergic neurons and enhances spinal 5-HT release; analgesia is reversed by 5-HT depletion, indicating descending pathway involvement.
Tazawa et al., 2015 [15]	Male Sprague–Dawley rats	Ligation of the left L5 spinal nerve after removal of the left L6 transverse process	3-hour stimulation: 50Hz pulses	Neuropathic Pain Model	SCS reverses SNL-induced mechanical hypersensitivity; intrathecal methysergide and idazoxan reduce the effect, indicating reliance on spinal serotonin and α2-adrenergic signaling; SCS increases DRN serotonergic activation and normalizes LC noradrenergic tone without elevating LC activity.
Koyama et al., 2018 [16]	Male Sprague–Dawley rats	Loose ligation of the common sciatic nerve	4-hour stimulation at 3-5Hz	Neuropathic Pain Model	SCS at sub-paresthesia intensity reverses thermal hyperalgesia and selectively reshapes S1 cortical oscillations, decreasing 3–4 Hz power, sparing 5–8 Hz theta, and increasing 10–30 Hz, supporting supraspinal cortical modulation accompanying analgesia.
Meuwissen et al., 2020 [17]	Male Sprague–Dawley rats	Unilateral ligation of the left sciatic nerve	1-hour stimulation: 50 Hz tonic, 449Hz burst	Neuropathic Pain Model	fMRI reveals that burst and tonic SCS differentially activate thalamus, S1, and ACC; burst engages limbic structures more, suggesting waveform-specific supraspinal processing.

To assess the risk of bias of the included studies, the SYRCLE RoB tool was utilized (Table 2). Sources of bias across 10 domains, including sequence generation, baseline characteristics, allocation concealment, random housing, blinding for performance bias, random outcome assessment, blinding for detection bias, incomplete outcome data, selective outcome reporting, and other potential sources of bias, were reviewed and assessed. Studies are organized by publication year.

**Table 2:** SYRCLE RoB assessment for the included seven studies SYRCLE RoB: Systematic Review Centre for Laboratory Animal Experimentation risk of bias.

Author (year)	Sequence generation	Baseline characteristics	Allocation concealment	Random housing	Blinding – performance bias	Random outcome assessment	Blinding – detection bias	Incomplete outcome data	Selective outcome reporting	Other sources of bias
Ren et al., 1996 [11]	No	Yes	No	No	No	No	No	Yes	Yes	Yes
Maeda et al., 2009 [12]	No	Yes	No	No	No	No	No	Yes	Yes	Yes
Barchini 2012 et al., [13]	No	Yes	No	No	No	No	No	Yes	Yes	Yes
Song et al., 2013 [14]	No	Yes	No	No	No	No	No	Yes	Yes	Yes
Tazawa et al., 2015 [15]	No	Yes	No	No	No	No	Yes	No	Yes	Yes
Koyama et al., 2018 [16]	No	Yes	No	No	No	No	No	Yes	Yes	Yes
Meuwissen 2020 et al., [17]	Yes	Yes	No	No	No	No	No	Yes	Yes	Yes

Discussion 

Spinal cord stimulation achieves analgesia through a coordinated interaction of segmental spinal inhibition and supraspinal modulation. Evidence from both electrophysiological recordings and lesion experiments demonstrates that SCS engages a bidirectional neuromodulatory feedback loop: stimulation of dorsal-column afferents inhibits nociceptive transmission in the dorsal horn, while ascending signals concurrently recruit brainstem centers that trigger descending inhibition, together reinforcing pain suppression. In this review, we identified seven key experimental studies that investigated these mechanisms in rodent models of neuropathic pain via peripheral nerve injuries. Although the designs and endpoints of the studies varied, collectively they support the concept that neither spinal nor supraspinal actions alone can fully account for SCS’s effects; instead, both levels are required for maximally effective analgesia.

While the SYRCLE risk-of-bias tool does not produce a numerical summary score, the number of “Yes” responses per study in this review ranged from 4 to 5, with an average of approximately 4.14 out of 11. This indicates a moderate to high risk of bias across the included studies and highlights the need for cautious interpretation of experimental findings. The variability in methodological rigor, including inconsistencies in randomization, blinding, and outcome reporting, may contribute to heterogeneity in observed outcomes. Mechanistic findings were extracted with attention to each study’s methodological context and interpreted in light of potential biases that may influence validity and translational relevance.

Segmental Spinal Inhibitory Mechanisms

A fundamental mechanism of SCS is the inhibition of pain signaling within the spinal dorsal horn. Several experiments demonstrate that SCS directly dampens pathological excitability in spinal nociceptive circuits. In a classic example, Ren et al. used a rat mononeuropathy model and showed that SCS selectively raised the early A-fiber-mediated flexor reflex threshold in the injured limb. The effect outlasted stimulation and persisted after mid-thoracic transection, indicating a segmental spinal mechanism [11]. Complementary microdialysis studies showed that SCS reduced dorsal-horn release of the excitatory amino acids glutamate and aspartate in neuropathic rats. This effect and the associated antihyperalgesia were diminished by GABAergic blockade, supporting a GABA-dependent segmental mechanism [18]. Alongside these results, Yakhnitsa et al. showed that SCS attenuated the hyper-responsiveness of dorsal horn neurons to tactile stimuli in neuropathic rats [19]. These findings support the gate-control theory: activation of large Aβ fibers by the SCS lead recruits inhibitory interneurons in the spinal dorsal horn, which gate incoming pain signals from C- and Aδ-fibers. Taken together, the included studies by Ren and colleagues indicate that segmental spinal mechanisms are a core component of SCS-induced analgesia. At the dorsal horn, SCS elevates reflex thresholds and attenuates neuropathy-related hyperexcitability, alongside reduced excitatory amino acid release and evidence of enhanced inhibitory transmission, consistent with a rebalancing of local excitatory/inhibitory drive. It is worth noting that SCS’s modulation of spinal processing may extend beyond neurons to glial and immune elements that contribute to central sensitization. Although not all included studies examined this aspect, recent research provides some insight. For instance, Liao et al. found that applying high-frequency SCS (10 kHz) in rats shortly after nerve injury prevented the upregulation of phosphorylated MAP kinases in the dorsal horn that is normally associated with the development of neuropathic pain [20]. This suggests that SCS can interrupt early signal transduction cascades involved in central sensitization. Another study by Shinoda et al. demonstrated that SCS attenuated microglial activation in the superficial dorsal horn following sciatic nerve injury [21]. Since activated microglia release pro-inflammatory mediators that enhance pain, SCS’s ability to suppress this response may further contribute to its segmental analgesic action. Although Shinoda et al. was not among the core seven studies, it reinforces the concept that neuroinflammatory modulation at the spinal level is another facet of SCS’s action.

Ascending Activation of Supraspinal Pathways

While SCS works at the spinal cord to gate pain, it simultaneously sends signals rostrally that activate supraspinal pain-modulating centers. Evidence from the reviewed studies highlights the importance of these ascending pathways. In the Barchini et al. rat neuropathy study, lesions of the dorsal columns to interrupt ascending input to the brain while still delivering SCS caudally attenuated the analgesic effect by approximately 50% [13]. Notably, Barchini et al. also demonstrated a similar ~50% loss of efficacy when descending pathways were disrupted, reinforcing that both directions are needed. Their pharmacological experiments further showed that blocking certain supraspinal-mediated receptors, such as 5-HT receptors, reduced SCS’s efficacy when stimulating above a dorsal column lesion, whereas blocking spinal GABA_A/GABA_B receptors was more impactful when stimulating below the lesion [13]. These results suggest that rostral supraspinal SCS effects rely on serotonergic mechanisms, whereas caudal segmental effects rely on GABAergic mechanisms. Maeda et al. reported that SCS at different frequencies induced c-Fos in discrete regions in a frequency-dependent manner [12]. In spared-nerve-injury (SNI) rats, 4 Hz evoked c-Fos in the nucleus raphe magnus, while the periaqueductal gray showed no change. Frequencies of 60 Hz and 100 Hz did not elicit supraspinal c-Fos at the sampled times. In uninjured controls, no supraspinal c-Fos changes were detected at the sampled times. This implies that the degree of supraspinal recruitment by SCS depends on stimulation parameters and injury state. Koyama et al., studying sub-perception SCS in a rat chronic constriction injury model, showed that low-frequency stimulation below motor threshold significantly reduced thermal hyperalgesia and normalized low-frequency theta-band oscillations in the somatosensory cortex, which is evidence of supraspinal modulation without overt paresthesia [16]. Meuwissen et al. used functional MRI in rats and found that burst and tonic SCS waveforms activated different brain regions: tonic SCS engaged the somatosensory cortex and thalamus, while burst SCS activated limbic regions, including the anterior cingulate cortex (ACC) and insula [17]. These results show that supraspinal mechanisms can be differentially recruited depending on the waveform used.

Recruitment of Descending Inhibitory Systems

Once the ascending drive from SCS reaches the brainstem, it engages descending inhibitory pathways. Song et al. recorded neuronal activity in the RVM and observed that SCS increased the firing of OFF-cells (antinociceptive) and decreased ON-cells (pronociceptive), but only in behavioral responders. Silencing the RVM via muscimol reduced the analgesic effect of SCS, demonstrating its necessity [14]. Song et al. also showed that SCS increased extracellular serotonin in the spinal dorsal horn and that blocking 5-HT receptors diminished analgesia [22]. Tazawa et al. examined both serotonergic and noradrenergic systems. SCS increased the activation of dorsal raphe nucleus serotonergic neurons, while locus coeruleus (LC) neurons showed no increase in phosphorylated cyclic AMP response element binding protein (pCREB). Intrathecal methysergide, a 5-HT1/2 antagonist, and idazoxan, an α2-adrenergic antagonist, each reduced the antihyperalgesic effect, indicating that spinal 5-HT and α2 signaling are required even without increased LC activation [15]. In another key study, Saadé et al. lesioned the dorsolateral funiculi (DLF), disrupting descending tracts, and found a 50% reduction in SCS analgesia [23], corroborating the dorsal column lesion results from Barchini et al. [13] and providing further evidence that both ascending and descending limbs are needed for SCS efficacy.

Integrated Feedback Loop

Bringing these mechanistic insights together, it becomes clear that effective SCS analgesia arises from both spinal and supraspinal contributions acting synergistically. Activation of dorsal-column afferents initiates local dorsal-horn gating, mediated by GABAergic and glycinergic interneurons and glial modulation, that suppresses aberrant nociceptive firing. Concurrently, ascending signals recruit brainstem and cortical circuits, engaging serotonergic and noradrenergic pathways as well as higher-order pain and affect centers. These supraspinal outputs then descend to reinforce spinal inhibition, creating a positive feedback loop that amplifies analgesia beyond what either segmental or supraspinal action alone could achieve. Recognizing this integrated loop helps bring together varied experimental findings and may guide refinement of clinical SCS parameters, such as waveform, frequency, and electrode placement, to better engage both limbs of the circuit and enhance patient outcomes.

## Conclusions

Although spinal cord stimulation has been extensively studied in preclinical settings, the field may benefit from further investigation into combined segmental and supraspinal mechanistic frameworks. By integrating data from seven key animal studies, this review aims to demonstrate that effective SCS analgesia arises from an interdependent spinal-supraspinal loop. At the spinal level, epidural stimulation helps restore inhibitory balance, enhancing GABAergic and glycinergic signaling while suppressing wide-dynamic-range neuron hyperactivity and mitigating neuroinflammatory cascades. Simultaneously, ascending afferent input recruits brainstem nuclei in the periaqueductal gray, rostroventromedial medulla, and locus coeruleus to initiate descending serotonergic and noradrenergic inhibition that further supports dorsal-horn gating. Waveform-specific paradigms, such as burst versus tonic stimulation, differentially engage cortical and limbic circuits, suggesting potential for more tailored neuromodulation strategies. Appreciating this dual-pathway mechanism may guide refinement of clinical SCS parameters, such as electrode placement, frequency, and stimulation pattern, to more effectively engage both limbs of the circuit and improve therapeutic outcomes. At the same time, these translational reviews should be balanced against the moderate risk of bias and species differences inherent in preclinical models. Future studies could employ more advanced approaches to deepen our understanding of this integrated network and support the development of next-generation, mechanism-driven SCS therapies for refractory neuropathic pain.

## Appendices

**Table 3:** Full search strategy utilizing the Cochrane Central Register of Controlled Trials. A comprehensive database of randomized and quasi‑randomized clinical trials compiled by the Cochrane Collaboration, drawing from MEDLINE, Embase, trial registries, and other sources to provide a centralized register of controlled studies for systematic reviews.

ID	Search
#1	MeSH descriptor: [Spinal Cord Stimulation] explode all trees
#2	MeSH descriptor: [Electric Stimulation] explode all trees
#3	MeSH descriptor: [Electric Stimulation Therapy] this term only
#4	((spine or spinal or transspinal or "trans spinal" or column* or epidural or lumbar or "dorsal column" or sacral or coccygeal or "conus medullari" or "conus medullaris" or "conus terminali" or "conus terminalis" or "medulla spinali" or "medulla spinalis" or myelon* or "thoracic cord" or "thoracic cords") NEAR/3 stimulat*)
#5	spinal cord stimulation or "spinal cord stimulations"
#6	(stimulat* NEAR/1 frequency)
#7	((therapy or therapies) NEAR/1 frequency)
#8	(burst NEAR/1 stimulat*)
#9	MeSH descriptor: [Electrodes, Implanted] explode all trees
#10	(electrode* NEAR/1 implant*)
#11	neuromodulat*
#12	#1 OR #2 OR #3 OR #4 OR #5 OR #6 OR #7 OR #8 OR #9 OR #10 OR #11
#13	MeSH descriptor: [Pain] explode all trees
#14	MeSH descriptor: [Pain Management] explode all trees
#15	(pain or pains):ti,ab,kw
#16	MeSH descriptor: [Nociceptors] explode all trees
#17	(nocicept*):ti,ab,kw
#18	#13 OR #14 OR #15 OR #16 OR #17
#19	#12 AND #18
#20	mechanis*
#21	#19 AND #20
#22	MeSH descriptor: [Models, Animal] explode all trees
#23	(animal* or rodent* or rat or rats or mouse or mice or "guinea pig" or "guinea pigs" or hamster* or rabbit* or cat or cats or dog or dogs or cattle or cow or cows or horse or horses or donkey* or mule or mules or monkey* or "nonhuman primate" or "non-human primate" or "nonhuman primates" or "non-human primates" or chimpanzee* or sheep or swine or pig or pigs or chicken or chick or chicks):ti,ab,kw
#24	#22 OR #23
#25	#21 AND #24

## References

[1] Deer TR, Grider JS, Lamer TJ (2020). A systematic literature review of spine neurostimulation therapies for the treatment of pain. Pain Med.

[2] Melzack R, Wall PD (1965). Pain mechanisms: a new theory. Science.

[3] Bentley LD, Duarte RV, Furlong PL, Ashford RL, Raphael JH (2016). Brain activity modifications following spinal cord stimulation for chronic neuropathic pain: a systematic review. Eur J Pain.

[4] Smits H, van Kleef M, Joosten EA (2012). Spinal cord stimulation of dorsal columns in a rat model of neuropathic pain: evidence for a segmental spinal mechanism of pain relief. Pain.

[5] Goudman L, De Groote S, Linderoth B, De Smedt A, Eldabe S, Duarte RV, Moens M (2021). Exploration of the supraspinal hypotheses about spinal cord stimulation and dorsal root ganglion stimulation: a systematic review. J Clin Med.

[6] Fang JY, Yamamoto H, Romman A, Koutrouvelis AP, Yamamoto S (2025). Spinal mechanisms of pain modulation by spinal cord stimulation: a systematic review. Cureus.

[7] Venna R, Yamamoto T, Romman A, Koutrouvelis AP, Yamamoto S (2025). Effectiveness of neuromodulation in postoperative pain management following spine surgery: a systematic review. Cureus.

[8] Yousaf A, Yamamoto H, Fang JY, Romman A, Koutrouvelis AP, Yamamoto S (2025). Supraspinal mechanisms of spinal cord stimulation in pain mitigation: a systematic review. Cureus.

[9] Yamamoto S, Duong A, Kim A (2023). Intraoperative spinal cord stimulation mitigates central sensitization after spine surgery in mice. Spine (Phila Pa 1976).

[10] Yamamoto S, Fang J, Eter A, Liu G, Nguyen A, Chung JM, La JH (2025). The role of NMDA and NK1 receptor signaling in spine surgery-induced central sensitization. Spine (Phila Pa 1976).

[11] Ren B, Linderoth B, Meyerson BA (1996). Effects of spinal cord stimulation on the flexor reflex and involvement of supraspinal mechanisms: an experimental study in mononeuropathic rats. J Neurosurg.

[12] Maeda Y, Ikeuchi M, Wacnik P, Sluka KA (2009). Increased c-fos immunoreactivity in the spinal cord and brain following spinal cord stimulation is frequency-dependent. Brain Res.

[13] Barchini J, Tchachaghian S, Shamaa F (2012). Spinal segmental and supraspinal mechanisms underlying the pain-relieving effects of spinal cord stimulation: an experimental study in a rat model of neuropathy. Neuroscience.

[14] Song Z, Ansah OB, Meyerson BA, Pertovaara A, Linderoth B (2013). The rostroventromedial medulla is engaged in the effects of spinal cord stimulation in a rodent model of neuropathic pain. Neuroscience.

[15] Tazawa T, Kamiya Y, Kobayashi A (2015). Spinal cord stimulation modulates supraspinal centers of the descending antinociceptive system in rats with unilateral spinal nerve injury. Mol Pain.

[16] Koyama S, Xia J, Leblanc BW, Gu JW, Saab CY (2018). Sub-paresthesia spinal cord stimulation reverses thermal hyperalgesia and modulates low frequency EEG in a rat model of neuropathic pain. Sci Rep.

[17] Meuwissen KP, van der Toorn A, Gu JW, Zhang TC, Dijkhuizen RM, Joosten EA (2020). Active recharge burst and tonic spinal cord stimulation engage different supraspinal mechanisms: a functional magnetic resonance imaging study in peripherally injured chronic neuropathic rats. Pain Pract.

[18] Cui JG, O'Connor WT, Ungerstedt U, Linderoth B, Meyerson BA (1997). Spinal cord stimulation attenuates augmented dorsal horn release of excitatory amino acids in mononeuropathy via a GABAergic mechanism. Pain.

[19] Yakhnitsa V, Linderoth B, Meyerson BA (1999). Spinal cord stimulation attenuates dorsal horn neuronal hyperexcitability in a rat model of mononeuropathy. Pain.

[20] Liao WT, Tseng CC, Wu CH, Lin CR (2020). Early high-frequency spinal cord stimulation treatment inhibited the activation of spinal mitogen-activated protein kinases and ameliorated spared nerve injury-induced neuropathic pain in rats. Neurosci Lett.

[21] Shinoda M, Fujita S, Sugawara S (2020). Suppression of superficial microglial activation by spinal cord stimulation attenuates neuropathic pain following sciatic nerve injury in rats. Int J Mol Sci.

[22] Song Z, Meyerson BA, Linderoth B (2011). Spinal 5-HT receptors that contribute to the pain-relieving effects of spinal cord stimulation in a rat model of neuropathy. Pain.

[23] Saadé NE, Barchini J, Tchachaghian S (2015). The role of the dorsolateral funiculi in the pain relieving effect of spinal cord stimulation: a study in a rat model of neuropathic pain. Exp Brain Res.

